# Neutrophil elastase plays a non‐redundant role in remodeling the venular basement membrane and neutrophil diapedesis post‐ischemia/reperfusion injury

**DOI:** 10.1002/path.5234

**Published:** 2019-03-22

**Authors:** Mathieu‐Benoit Voisin, Giovanna Leoni, Abigail Woodfin, Laure Loumagne, Nimesh SA Patel, Rosanna Di Paola, Salvatore Cuzzocrea, Christoph Thiemermann, Mauro Perretti, Sussan Nourshargh

**Affiliations:** ^1^ William Harvey Research Institute, Barts and The London School of Medicine and Dentistry Queen Mary University of London London UK; ^2^ Institute for Cardiovascular Prevention (IPEK) Ludwig‐Maximilian University (LMU) Munich Germany; ^3^ Department of Chemical, Biological, Pharmaceutical and Environmental Sciences University of Messina Messina Italy

**Keywords:** neutrophil, elastase, ischemia/reperfusion injury, venular basement membrane

## Abstract

Ischemia/reperfusion (I/R) injury is a severe inflammatory insult associated with numerous pathologies, such as myocardial infarction, stroke and acute kidney injury. I/R injury is characterized by a rapid influx of activated neutrophils secreting toxic free radical species and degrading enzymes that can irreversibly damage the tissue, thus impairing organ functions. Significant efforts have been invested in identifying therapeutic targets to suppress neutrophil recruitment and activation post‐I/R injury. In this context, pharmacological targeting of neutrophil elastase (NE) has shown promising anti‐inflammatory efficacy in a number of experimental and clinical settings of I/R injury and is considered a plausible clinical strategy for organ care. However, the mechanisms of action of NE, and hence its inhibitors, in this process are not fully understood. Here we conducted a comprehensive analysis of the impact of NE genetic deletion on neutrophil infiltration in four murine models of I/R injury as induced in the heart, kidneys, intestine and cremaster muscle. In all models, neutrophil migration into ischemic regions was significantly suppressed in NE^−/−^ mice as compared with wild‐type controls. Analysis of inflamed cremaster muscle and mesenteric microvessels by intravital and confocal microscopy revealed a selective entrapment of neutrophils within venular walls, most notably at the level of the venular basement membrane (BM) following NE deletion/pharmacological blockade. This effect was associated with the suppression of NE‐mediated remodeling of the low matrix protein expressing regions within the venular BM used by transmigrating neutrophils as exit portals. Furthermore, whilst NE deficiency led to reduced neutrophil activation and vascular leakage, levels of monocytes and prohealing M2 macrophages were reduced in tissues of NE^−/−^ mice subjected to I/R. Collectively our results identify a vital and non‐redundant role for NE in supporting neutrophil breaching of the venular BM post‐I/R injury but also suggest a protective role for NE in promoting tissue repair. © 2019 The Authors. *The Journal of Pathology* published by John Wiley & Sons Ltd on behalf of Pathological Society of Great Britain and Ireland.

## Introduction

Ischemia/reperfusion (I/R) injury is a common feature of many cardiovascular pathologies, such as myocardial infarction (MI), cerebral stroke and clinical complications associated with organ transplantation surgeries 1, 2, 3, 4. The occurrence of I/R injury is paradoxical in that it is induced following the often life‐saving process of restoration of blood flow to ischemic tissues. Although this practice is aimed at providing nutrients and oxygen to affected regions and, hence, preventing tissue necrosis, the procedure can lead to vascular dysfunction 5, acute inflammation and ultimately an increase in tissue cell death 6. This pathophysiological response is largely the consequence of a rapid activation of the vascular endothelium and an intense recruitment of proinflammatory leukocytes 7.

Neutrophils are the first leukocytes to be recruited to sites of I/R injury. These innate immune cells, whilst vital for host defense against pathogens, are implicated in the pathogenesis of many disorders due to their capacity to release a wide range of proinflammatory mediators 8, 9, reactive oxygen species (ROS) 10 and destructive proteolytic enzymes. Clear evidence for the involvement of neutrophils in I/R injury has been provided by both preclinical and clinical studies in which neutrophil depletion was protective against further tissue damage 11, 12, 13, 14. Similarly, blocking neutrophil interaction with blood vessel walls within reperfused areas of ischemic tissues improves disease outcome in patients 15, 16, 17. However, the inhibitors used in such settings are not solely specific for neutrophil recruitment and also blocked the migration of other leukocyte subtypes, including monocytes that can contribute to tissue healing. As such, there remains a need for alternative therapeutic approaches for suppressing neutrophil migration and their destructive potential following I/R injury.

Neutrophil elastase (NE) is the most abundant protease expressed by neutrophils 18, 19 and is rapidly released from azurophilic granules upon activation. This serine protease can act on a broad range of substrates, including extracellular matrix (ECM) components, proenzymes, adhesion molecules, signaling receptors and cytokines 20, 21. It also has strong antibacterial properties and is implicated in NETosis 22. Due to this wide‐ranging substrate specificity and function, NE has been implicated in numerous physiological and pathological scenarios, including I/R injury 23, and is considered to be a good indicator of disease severity in respiratory and cardiovascular pathologies 24, 25. Interestingly, natural and synthetic NE blockers inhibited I/R‐induced inflammation in both preclinical and clinical studies 26. In sharp contrast, NE‐deficient animals (NE^−/−^) showed normal neutrophil recruitment in experimental models of cytokine‐induced inflammation 27 and bacterial infection 28, 29. Taken together, such conflicting studies suggest a stimulus‐specific role for NE as a regulator of neutrophil trafficking and, importantly, highlight the lack of understanding of the mechanisms through which NE regulates neutrophil migration and possibly activation.

The aim of the present study was to re‐evaluate the potential strength of NE as a therapeutic target for the suppression of I/R‐induced inflammation and to ascertain its mechanism of action in mediating neutrophil trafficking post‐I/R injury. We hypothesized that during I/R injury, NE is rapidly induced on the cell surface of transmigrating neutrophils and, as such, plays a unique and non‐redundant role in facilitating neutrophil migration through blood venular walls. For this purpose, we investigated the role of NE using both NE‐deficient mice and a well‐characterized NE inhibitor (ONO‐5046, sivelestat) 30, in multiple disease‐mimicking murine models of I/R injury, including models of myocardial and renal I/R injury, and have ascertained the site of arrest of neutrophils at the level of the venular basement membrane (BM) under conditions of NE deletion/blockade during I/R injury. Mechanistically, the function of NE was linked to its mobilization from transmigrating neutrophils within the venular BM and its ability to remodel neutrophil permissive regions within the venular BM termed low expression regions (LERs). Overall, the present findings provide unequivocal evidence for an essential role for NE in neutrophil migration through venular walls in I/R injury, identifying the breaching of the venular BM as a key NE‐mediated stage of neutrophil trafficking during this pathology.

## Materials and methods

Detailed materials and methods are available in supplementary material, Supplementary materials and methods.

### Animals

NE knockout (NE^−/−^) male mice and C57BL/6 wild‐type (WT) animals were employed. All animal experiments were conducted in accordance with the UK Home Office legislation and NC3R recommendations.

### Treatments

Anesthetized WT mice were injected via jugular vein cannulation with saline or with sivelestat (ONO‐5046) with an initial dose of 50 mg/kg in a 200 μl bolus followed by an infusion of 50 mg/kg at 200 μl/h.

### I/R injury of the heart

Anesthetized animals were subjected to MI by ligation of the left anterior descending coronary artery for 25 min followed by a reperfusion period of 2 h.

### I/R injury of the kidneys

Anesthetized animals were subjected to bilateral renal ischemia for 30 min followed by a 24 h reperfusion period.

### Intravital microscopy (IVM) and induction of cremasteric I/R injury

The cremaster muscle was surgically exteriorized and subjected to ischemia using an artery clamp placed at the proximal end of the cremaster tissue for 30 min. Blood flow was then restored by releasing the clamp and reperfusion was allowed to develop for up to 2 h. Leukocyte responses (adhesion and extravasation) were observed on an upright brightfield microscope.

### IVM and induction of I/R injury of the mesenteric tissue

Mesenteric ischemia was induced by clamping the superior mesenteric artery for 35 min before allowing reperfusion of the tissue for 90 min. Leukocyte responses (adhesion and extravasation) were quantified within mesenteric post‐capillary venules.

### Determination of renal injury and dysfunction

Histological evaluation was performed on kidney sections stained with H&E and viewed using a brightfield microscope. The serum levels of creatinine and aspartate aminotransferase were quantified by ELISA.

### Adoptive cell transfer experiments

Bone marrow leukocytes from WT or NE^−/−^ donor mice were fluorescently labeled with PKH26 according to the manufacturer's recommendations and injected i.v. into WT or NE^−/−^ recipient animals via the tail vein prior to I/R injury of the cremaster muscles as detailed above. At the end of the reperfusion period, the harvested tissues were immunostained for confocal microscopy analysis. Blood samples were also taken by cardiac puncture and the number of circulating PKH26+ cells was analyzed by flow cytometry.

### Analysis of tissues by immunofluorescence labeling and confocal microscopy

Detection of neutrophils into the ischemic region of the heart was performed on OCT‐embedded heart tissue sections immunostained for collagen IV, endothelial cells and neutrophils. For localization of neutrophils into the cremaster muscles and mesenteric tissue following I/R injury, whole‐mount tissues were fluorescently immunostained for neutrophils, the endothelium and the perivascular BM. For NE expression, tissues were fluorescently immunostained for neutrophils, NE and the perivascular BM. For NE activity, the NE‐fluorescent activatable substrate NE680FAST (Perkin Elmer, Beaconsfield, UK) was injected i.v. prior to reperfusion. All samples were viewed using laser scanning confocal microscopes and images were analyzed using IMARIS software. The area and intensity of matrix protein LERs within the perivascular BM were measured using ImageJ (NIH & Laboratory for Optical and Computational Instrumentation, University of Wisconsin, Madison, WI, USA).

### Vascular leakage

Vascular leakage upon I/R injury was quantified using the Miles assay 31.

### Flow cytometry analysis of leukocyte subpopulation

Leukocyte subpopulations (neutrophils, monocytes and macrophages) present in the cremaster muscles and their phenotype were assessed by flow cytometry 20 h post‐I/R injury.

### Statistical analyses

All data were processed and analyzed using GraphPad Prism 6 software (GraphPad Inc, San Diego, CA, USA) and are presented as mean ± SEM. Statistical significance was assessed by parametric or non‐parametric tests according to the sample size of the data analyzed. *p* < 0.05 was taken as statistically significant.

## Results

### NE‐deficient mice exhibit reduced neutrophil infiltration in models of myocardial and kidney I/R injury

**Figure 1:**
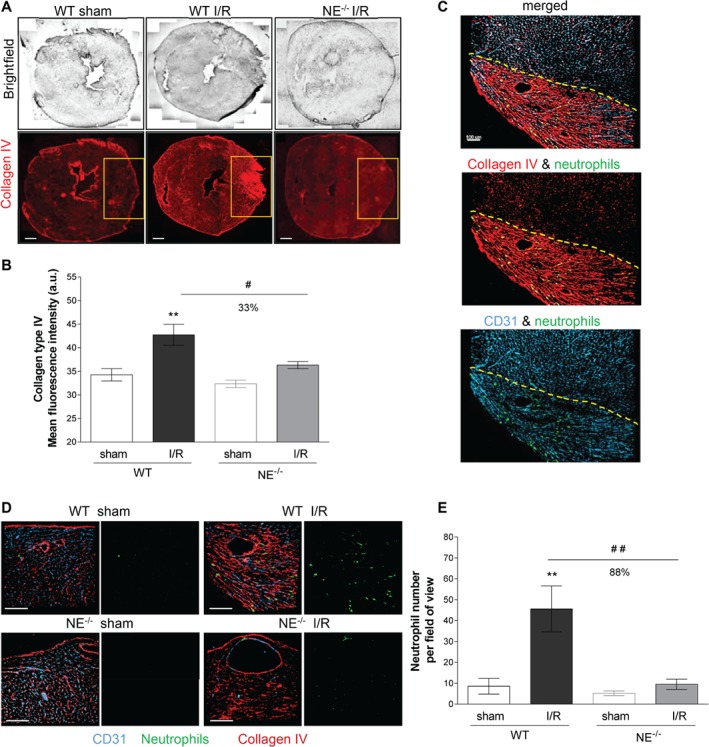
Neutrophil recruitment into the area at risk of NE‐deficient mice (NE^−/−^) is impaired following MI and reperfusion injury. (A) Reconstructed tiling images of myocardial cryosections acquired by confocal microscopy from WT animals and NE^−/−^ mice and subjected to 25 min of MI followed by 2 h of reperfusion (I/R). Tissue sections from sham‐operated WT (left panels), I/R‐subjected WT (middle panels) and I/R‐subjected NE^−/−^ (right panels) animals were immunostained for collagen type IV (red). The yellow boxes exemplify the ischemic and reperfused regions of the left ventricle (area at risk). (B) Quantification of the collagen type IV mean intensity in the area at risk (yellow box from (A)). (C) Confocal images of the area at risk (delimited by the dotted line) from a WT animal subjected to myocardial I/R and immunostained for collagen type IV (red), PECAM‐1 (blue) and MRP‐14 (green) to visualize the ECM, blood vasculature and neutrophils, respectively. The image shows neutrophils infiltrating the region at risk only. (D) Magnified regions of the region of left ventricle subjected or not to I/R from WT (top panels) and NE^−/−^ (bottom panels) animals. Cryosections were immunostained for collagen type IV (red), PECAM‐1 (blue) and MRP‐14 (green) to visualize the ECM, blood vasculature and neutrophils, respectively. Left panels show the three channels together, whereas the right panels show the neutrophil channel only. The images exemplify the absence of neutrophil infiltration in the region at risk of the myocardium from NE^−/−^ mice subjected to I/R as compared with WT littermates. The top right panels (WT I/R) are magnified images of the same sample region as shown in (D). (E) Quantification of the number of neutrophils present in the area at risk. Data represent mean ± SEM from three or four mice per group. **p < 0.01 for the comparison between I/R and sham‐operated animals; #*p* < 0.05, ##*p* < 0.01 for the comparison between WT and NE^−/−^ mice as indicated by lines. Scale bars = 100 μm.

To ascertain the role of NE in neutrophil recruitment in clinically relevant models of I/R injury we investigated the impact of genetic deletion of NE in murine models of myocardial and kidney I/R injury. With respect to the heart injury model, WT and NE^−/−^ animals were subjected to MI for 25 min followed by 2 h of reperfusion. The degree of tissue injury (depicted by increased anti‐collagen mAb immunoreactivity) and levels of neutrophil infiltration into the heart ischemic region were investigated by confocal microscopy. WT mice subjected to MI exhibited an intense remodeling of the myocardial tissue, as illustrated by increased collagen IV immunoreactivity in the ischemic area (Figure 1A,B), a response that was associated with a significant infiltration of neutrophils specifically in this region (Figure 1C) as compared with sham‐operated WT mice (Figure 1D,E). In contrast, NE^−/−^ animals subjected to MI exhibited a similar morphology and levels of collagen IV immune reactivity to that seen in sham‐operated animals (Figure 1A,B). Interestingly, NE^−/−^ mice exhibited no increase in local neutrophil infiltration post‐MI as compared with WT animals (Figure 1D,E).

**Figure 2:**
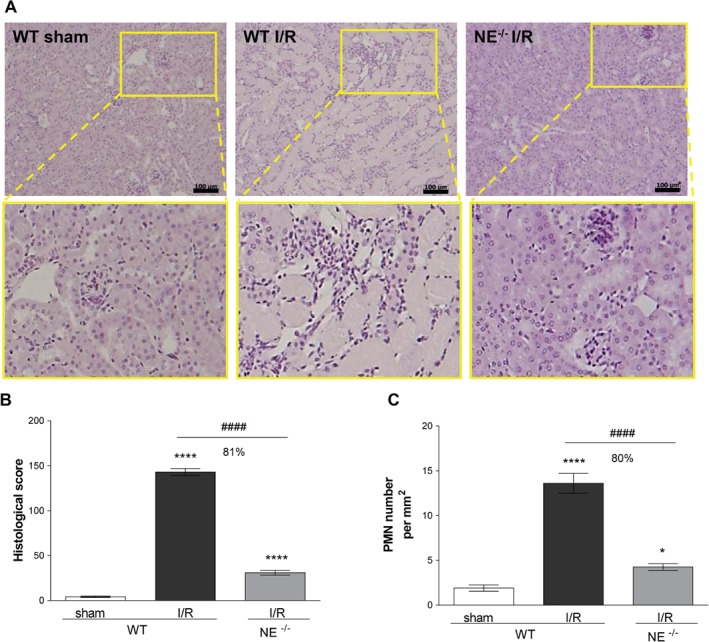
NE‐deficient mice are protected from renal I/R injury. (A) Histopathology of kidney sections from sham‐operated WT or WT and NE^−/−^ mouse subjected to renal I/R and stained with H&E. The bottom pictures are magnified regions (yellow box) demonstrating the modification of the architectural structure of tubules and glomeruli upon I/R injury in WT but not NE^−/−^ animals. (B) Histological score analysis of kidney sections. (C) Quantification of the number of PMN infiltrating the kidney. Data represent mean ± SEM from at least four animals per group. *p < 0.05 and ****p < 0.0001 for the comparison between I/R and sham‐operated animals; ####*p* < 0.0001 for the comparison between WT and NE^−/−^ mice as indicated by lines. Scale bar = 100 μm.

To ascertain the generality of the protective effect of NE genetic deficiency against I/R injury and neutrophil infiltration, we next investigated a model of renal I/R injury. Here, mice were subjected to bilateral renal ischemia for 30 min followed by reperfusion for 24 h before collecting kidney and blood samples to assess the extent of local tissue damage, leukocyte infiltration and the loss of renal functions post‐I/R. Histological examination of WT kidneys subjected to I/R showed evidence of renal injury, as exemplified by the degeneration of proximal and distal tubules, tubular swelling and necrosis, and luminal congestion, as compared with sham‐operated animals (Figure 2A,B). This response was also associated with increased polymorphonuclear cell (PMN) infiltration (Figure 2C, supplementary material, Figure S1A). In contrast, NE^−/−^ animals subjected to renal I/R showed almost normal kidney structure organization and an approximately 80% reduction in leukocyte infiltration**.** Analysis of sera from WT mice subjected to renal I/R showed a significant increase in markers of renal dysfunction/injury, namely high levels of creatinine and aspartate aminotransferase, as compared with levels detected in the serum of both sham‐operated WT mice and NE^−/−^ animals subjected to kidney I/R (Figure 1B,C).

Collectively these results demonstrate that NE deficiency is protective in both models of MI and renal I/R injury, an effect that involved a marked inhibition of the acute neutrophil recruitment to locally injured tissues.

### NE deficiency inhibits I/R‐induced neutrophil migration into tissues by selectively suppressing breaching of venular walls at the level of the BM

**Figure 3:**
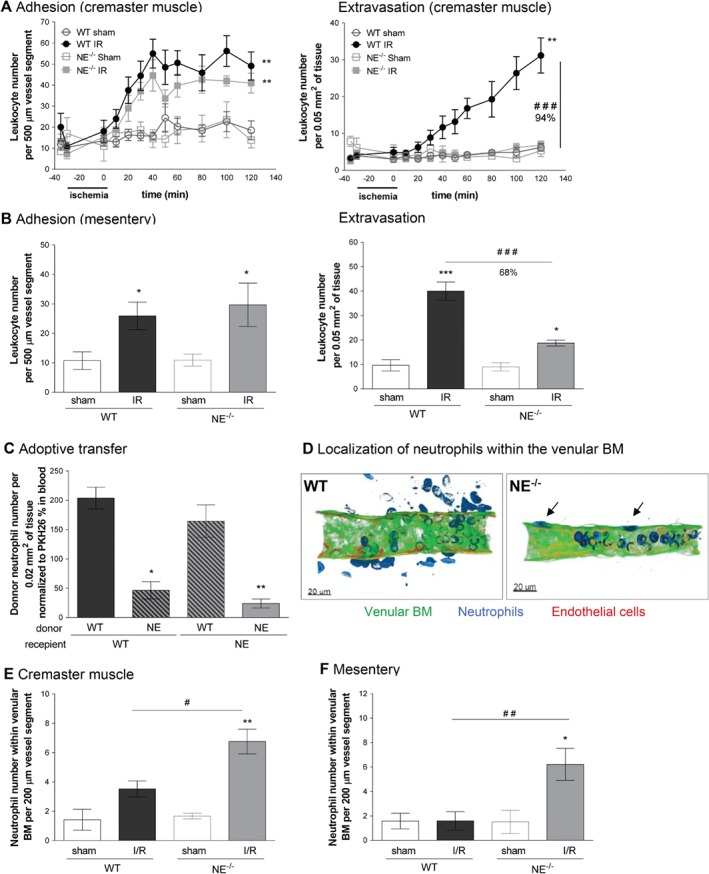
Effect of NE deficiency on leukocyte transmigration responses in post‐capillary venules *in vivo* following I/R injury. Leukocytes' firm adhesion and transmigration in post‐capillary venules of mouse cremaster muscles or mesentery in response to I/R were investigated using IVM. (A) The mouse cremaster muscle from WT and NE^−/−^ animals was surgically exteriorized, superfused with Tyrode's solution and basal leukocyte responses were quantified for 20 min prior to the induction of ischemia using a clamp, as detailed in Materials and methods. Thirty minutes later, the clamp on the exteriorized cremaster muscle was removed to induce reperfusion of the vessels. Leukocyte adhesion (left panel) and extravasation (right panel) responses were quantified at regular intervals for 120 min post‐reperfusion. For each genotype, a sham‐operated group was also analyzed (exteriorized cremaster tissues without the clamp). (B) WT and NE^−/−^ mice were subjected to occlusion of the superior mesenteric artery for 35 min, followed by 90 min reperfusion. For each genotype, a sham‐treatment group was also analyzed, where laparotomy was conducted without the occlusion of the mesenteric arteries. Both leukocyte adhesion (left panel) and transmigration (right panel) responses were quantified in post‐capillary venules at 90 min post‐reperfusion. (C) Bone marrow neutrophils were isolated from WT or NE^−/−^ mice, fluorescently labeled and injected i.v. into WT or NE−/− recipient mice prior to the induction of I/R of the cremaster muscle, as detailed in Materials and methods. At the end of the experiment, tissues where harvested, fixed and immunostained for neutrophils (MRP‐14). Data show the quantification of fluorescent donor neutrophils present in the tissue and normalized to the number of blood circulating donor cells. (D) The images are representative confocal pictures of post‐capillary venules of the cremaster muscles of WT (left panel) and NE^−/−^ (right panel) mice subjected to 30 min ischemia followed by 120 min reperfusion of the cremaster muscles. The images show that whereas in WT neutrophils access the interstitial tissue, NE^−/−^ cells are trapped within the venular BM (arrows). (E) Quantification of the number of neutrophils present within the venular BM 2 h post‐reperfusion of the cremaster muscles. (F) Quantification of the number of neutrophils present within the venular BM following 1.5 h post‐reperfusion of the mesentery. Figures are representative of four to seven animals per group. Mean ± SEM. *p < 0.05, **p < 0.01, ***p < 0.001 for the comparison between I/R and sham‐operated animals (or between WT and NE^−/−^ donor cells for the adoptive transfer experiment); #*p* < 0.05, ##*p* < 0.01, ###*p* < 0.001 for the comparison between WT and NE^−/−^ mice as indicated by lines. Bars = 20 μm.

To gain insight into the mechanism through which NE mediates I/R‐induced neutrophil recruitment, we sought to determine the stage of the emigration cascade at which NE acts. For this purpose, we first applied IVM to the analysis of leukocyte responses within cremasteric venules of WT and NE^−/−^ mice subjected to local I/R injury, as previously described 32, 33. Within this I/R model, robust and time‐dependent leukocyte adhesion and transmigration responses were noted within the 2 h reperfusion period in WT mice, as compared with sham‐operated animals (Figure 3A, supplementary material, Movies S1,S2). Interestingly, neutrophil emigration into inflamed tissues was almost completely inhibited in NE^−/−^ animals (94% inhibition), whereas leukocyte adhesion was unaffected (Figure 3A). Of note, the percentage of blood circulating neutrophils and key microvascular flow hemodynamic parameters were the same between WT and NE^−/−^ mice (see supplementary material, Figure S2). Pretreatment of WT animals with silvestat also resulted in an inhibition of leukocyte extravasation but not adhesion (Figure 3A,B). Similar results were obtained in a model of mesenteric I/R where NE^−/−^ mice showed no defect in leukocyte adhesion but were profoundly suppressed in terms of leukocyte extravasation into interstitial tissues as compared with WT animals (Figure 3B, supplementary material, Figure S2, Movie S3).

To ascertain if the role of NE in mediating neutrophil breaching of venular walls is driven by a cell‐autonomous mechanism involving neutrophil‐derived NE (as opposed to tissue‐derived NE), we conducted a series of cell transfer experiments in which fluorescently labeled bone marrow leukocytes derived from WT and NE^−/−^ mice were injected i.v. into WT or NE^−/−^ recipient animals prior to I/R injury. At the end of the reperfusion period, the transmigration response of donor leukocytes in cremaster muscles was analyzed by confocal microscopy. Donor WT leukocytes showed similar profiles of transmigration post‐I/R injury when injected in WT or NE^−/−^ recipient animals, suggesting that the genetic background of the recipient mouse (i.e. NE deficiency in the vasculature and interstitial tissue) does not account for the defective neutrophil transmigration response noted in NE^−/−^ animals (Figure 3C). However, when NE^−/−^ leukocytes were transferred into WT or NE^−/−^ recipient animals, the migration of these donor cells into tissues was significantly suppressed, indicating that the role of NE in the migration of neutrophils through the vessel wall in I/R injury is a cell‐autonomous effect.

As breaching the venular wall involves penetrating multiple barriers, including the endothelium and the venular BM 34, 35, we next determined the site of arrest of NE‐deficient neutrophils within venular walls in whole‐mount immunostained tissues using confocal microscopy. The images showed a significant increase in the number of neutrophils that had breached the endothelium but were retained within the venular wall in NE^−/−^ mice as compared with WT controls post‐reperfusion period (Figure 3D,E). Similar results were obtained in the model of mesenteric I/R (Figure 3F). WT mice pretreated with the NE inhibitor sivelestat and subjected to cremasteric I/R also showed an increased number of neutrophils within the venular wall post‐breaching of the endothelium, as compared with mice treated with the drug vehicle (see supplementary material, Figure S3C).

Collectively these results identify a selective role for neutrophil‐derived NE in mediating leukocyte migration through venular walls, a response that appears to occur in a cell‐autonomous manner. Furthermore, although NE deletion or pharmacological blockade does not impact neutrophil transendothelial migration, NE appears to play an important role in neutrophil breaching of the venular BM post‐I/R injury.

### Neutrophil‐derived NE is retained within the venular BM during neutrophil transmigration

**Figure 4:**
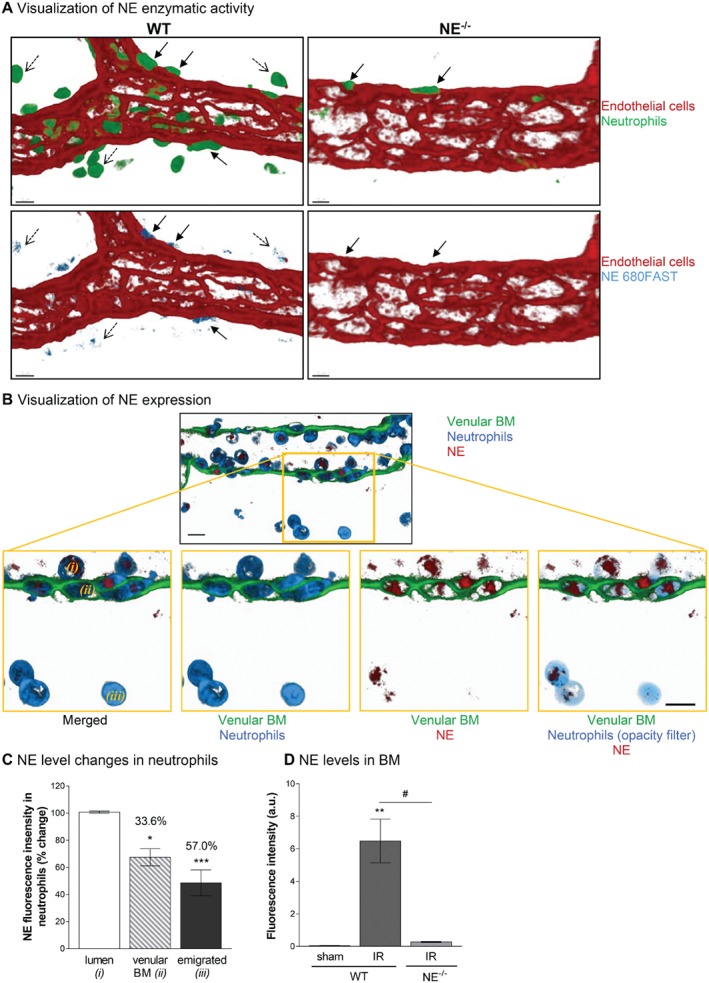
NE is mobilized during the migration of neutrophils through the venular BM. The cremaster muscles and mesentery of WT and NE^−/−^ mice were subjected to temporary ischemia followed by reperfusion. At the end of the reperfusion period, tissues were collected, fixed and immunostained prior to visualization of the samples using confocal microscopy. (A) Representative image of a post‐capillary venule (CD31, red) of WT and NE^−/−^ animals subjected to I/R injury and demonstrating the association of the enzymatic activity of NE using the NE680FAST fluorescent substrate (blue) with neutrophils (green) present in the abluminal aspect (i.e. BM) of the vessel wall (plain arrows) and, to a lesser extent, interstitial neutrophils (dotted arrows). (B) Representative confocal image of a post‐capillary venule (2 μm longitudinal cross‐section from the middle of the vessel, green) from a WT mouse subjected to I/R showing NE expression (red) in the cytoplasmic compartments (blue) of neutrophils. The bottom panels are enlargements of the yellow boxed region showing neutrophils at different stages of their migration route, i.e. luminal (*i*), within the vascular BM (*ii*) or within the interstitial tissue (*iii*). A 5% opacity filter on the MRP‐14 channel was applied to highlight NE expression in neutrophils on the right panel. (C) Quantification of the mean fluorescence intensity of NE expression in neutrophils at the three stages of their migration (as expressed as a percentage change over the intensity of NE from luminal neutrophils). (D) Quantification of the fluorescence intensity of NE expression within the venular BM from WT and NE^−/−^ animals at 1 h post‐reperfusion. Figures are representative of four to seven animals per group. Mean ± SEM. *p < 0.05, **p < 0.01, ***p < 0.001, for the comparison of NE intensity (mean fluorescence intensity) between BM/interstitial neutrophils and luminal cells (C) or between I/R and sham‐operated animals (D); #*p* < 0.05 for the comparison between WT and NE^−/−^ mice as indicated by the line. Bars = 10 μm.

Having found that NE deficiency leads to neutrophil retention at the level of the venular BM in a cell‐autonomous manner, we aimed to gain more insight into the mechanism through which this happens. Initially, we determined the localization of NE enzymatic activity and protein expression during the process of neutrophil migration from the vascular lumen into the interstitial space as assessed by immunostaining and analysis of tissues by confocal microscopy. Using a specific NE‐fluorescent activatable substrate (NE680FAST) injected i.v., we observed an intense NE activity associated with neutrophils located in the abluminal aspect of the vessel wall (i.e. within the venular BM), and at a lower level in tissue‐infiltrated leukocytes (Figure 4A). Similarly, using a specific Ab (see supplementary material, Figure S4), NE was strongly associated with neutrophils within both the vascular lumen and the venular wall, and, to a lesser extent, in tissue‐infiltrated neutrophils (Figure 3B,C). Quantification of neutrophil NE expression at different stages of neutrophil migration through the venular wall demonstrated a significant decrease in the neutrophil‐associated NE as the cells moved from the vascular lumen to the interstitial tissue. Specifically, neutrophils within the venular BM and interstitial tissues post‐I/R injury showed 33.6 and 57.0% reduction in NE expression, as compared with luminal neutrophils, respectively. Interestingly, this response was associated with an increase in NE localization within the BM itself in WT mice subjected to I/R injury but not in NE^−/−^ animals (Figure 3D).

Taken together, these results suggest that during I/R injury neutrophils release approximately one third of their NE content within the venular BM as they encounter this structure during their emigration into the interstitium.

### NE mediates remodeling of the venular BM

**Figure 5:**
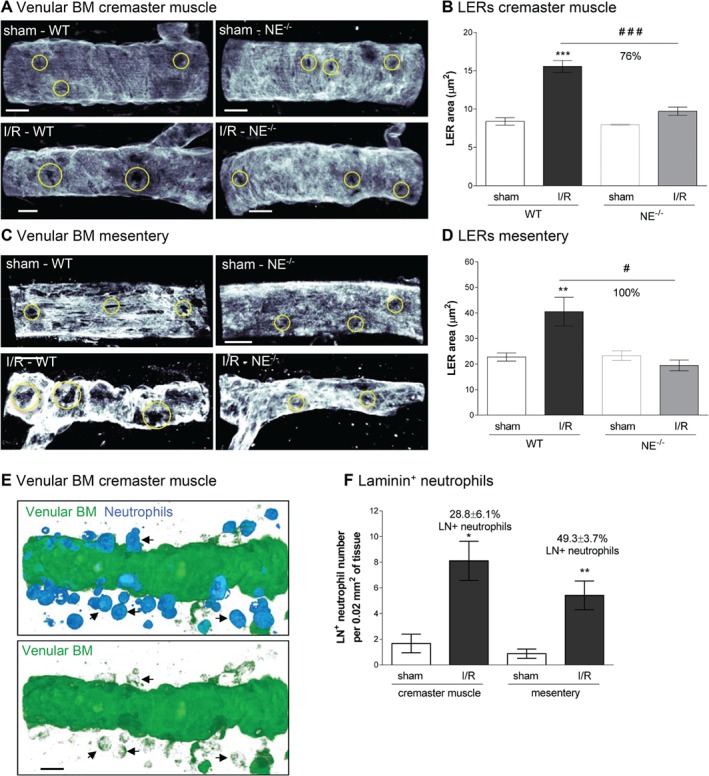
Venular BM is remodeled during I/R injury and transmigrated neutrophils are positive for laminin. WT and NE^−/−^ mice were subjected to I/R of their cremaster muscles or mesentery as described in Materials and methods. At the end of the reperfusion period, tissues were collected, fixed and whole‐mount immunostained for neutrophils (MRP‐14) and BM (laminin‐α5 or pan‐laminin) prior to visualization of the samples by confocal microscopy. (A) Representative images showing the presence of LERs (circles) within the BM (laminin‐α5) of post‐capillary venules of mouse cremaster muscles. (B) Quantification of the size of the LER within the BM (laminin‐α5) of the cremaster post‐capillary venules. (C) Representative images showing the presence and remodeling of LERs (circles) within the BM (laminin‐α5) of post‐capillary venules of mouse mesentery. (D) Quantification of the size of the LER within the BM (laminin‐α5) of the mesenteric post‐capillary venules. (E) Representative confocal image of a post‐capillary venule (from a WT mouse subjected to I/R) showing the presence of laminin‐positive neutrophils within the interstitial tissue (arrows). The bottom picture shows the staining of laminin only. (F) Quantification of the number of neutrophils present within the venular BM 1.5 h post‐reperfusion of the mesentery. Figures are representative of four to seven animals per group. Mean ± SEM. *p < 0.05**,** **p < 0.01, ***p < 0.001, for the comparison between sham and I/R groups; #*p* < 0.05, ###*p* < 0.001 for the comparison between WT and NE^−/−^ mice as indicated by the line. Bars = 10 μm.

We have previously reported on the expression and remodeling of regions within the venular BM that exhibit reduced levels of certain matrix proteins (e.g. collagen IV and laminins) that act as preferred exit portals for transmigrating neutrophils 36, 37. These regions, termed matrix protein LERs, were investigated here in terms of their size by confocal microscopy in cremasteric and mesenteric tissues post‐I/R injury. Although WT mice subjected to I/R showed an increased average size of venular BM LERs as compared with sham‐treated mice, no such increase was noted in NE^−/−^ animals (Figure 5A–D). Pharmacological blockade of NE also suppressed the increase in size of LERs in the cremaster muscle I/R injury model (see supplementary material, Figure S3D). Interestingly, immunostaining of WT tissues with Abs against neutrophils and venular BM (pan‐laminin Ab), demonstrated that approximately 30 and 50% of tissue‐infiltrated neutrophils were immunoreactive for laminin in the cremaster muscle and mesenteric I/R models, respectively (Figure 5E,F), suggesting carriage of laminin fragments by migrating neutrophils.

Taken together these results demonstrate that NE can facilitate remodeling of the venular BM through increasing the size of neutrophil permissive regions. This remodeling probably occurs through disruption of the laminin network of the BM, as mediated via cleavage and carriage of laminin fragments by the emigrating neutrophils.

### NE^−/−^ mice exhibit reduced I/R‐induced neutrophil activation and vascular leakage, but also show suppressed recruitment of monocytes and prohealing M2 macrophages in inflamed tissues

**Figure 6:**
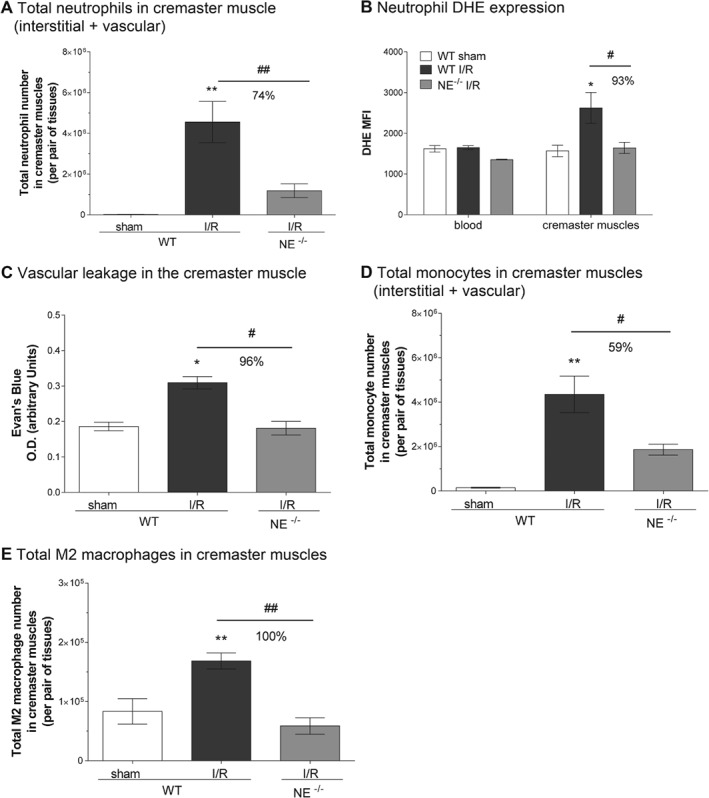
NE genetic deficiency inhibits neutrophil activation and monocyte/macrophage recruitment. The cremaster muscles of WT and NE^−/−^ mice were subjected to temporary ischemia followed by reperfusion; leukocyte phenotype and vascular leakage were quantified by flow cytometry and Evan's Blue assay, respectively. (A) Quantification of the total number of neutrophils in the cremaster muscles (i.e. interstitial and vascular neutrophils included). (B) Quantification of ROS generation by isolated neutrophils from blood or cremaster muscles as measured by DHE mean fluorescence intensity (MFI). (C) Quantification of the vascular leakage into the tissue post‐reperfusion (4 h). (D) Quantification of the total number of monocytes in the cremaster muscles at 20 h post‐reperfusion. (E) Quantification of the total number of M2 (CD206+) macrophages in the cremaster muscles at 20 h post‐reperfusion. Data represent mean ± SEM from four to six mice per group (from three independent experiments). *p < 0.05, **p < 0.01, for the comparison between sham and I/R groups; #*p* < 0.05, ##*p* < 0.01 for the comparison between WT and NE^−/−^ mice as indicated by the line.

In a final series of experiments we investigated the potential impact of NE deletion on other key cellular features of I/R injury, namely neutrophil activation, vascular leakage and recruitment of monocytes. The activation state of tissue‐infiltrated neutrophils (both in the interstitium and vasculature; Figure 6A) in sham and I/R‐injured cremaster muscles of WT and NE‐deficient mice was quantified through analyzing levels of ROS by flow cytometry. Although neutrophils present in WT cremaster muscles exhibited an enhanced dihydroethidium (DHE) signal (Figure 6B) (and higher CD11b expression, MFI = 21 732 ± 4389 versus 14 276 ± 1335 for I/R versus sham‐operated group, respectively) as compared with cells in sham‐operated tissues or blood leukocytes, the few neutrophils found in NE^−/−^ tissues (mainly within the vasculature and the venular wall (Figures 3, 6A) had a reduced DHE signal (Figure 6A,B). The suppressed activation state of NE^−/−^ tissue neutrophils was associated with reduced vascular leakage in the knockout mice post‐I/R injury, as compared with the response noted in WT animals (Figure 6C). Interestingly, however, in assessing the impact of NE deficiency on the resolution phase of the I/R injury, although both monocyte and M2 macrophage numbers were elevated in WT cremaster muscles at 20 h post‐reperfusion (Figure 6D,E), these responses were markedly inhibited in NE^−/−^ animals. Of note, local I/R insult had no impact on blood monocyte numbers in either WT or NE^−/−^ animals (see supplementary material, Figure S5).

Collectively, these results suggest that although blocking NE‐dependent neutrophil extravasation may be an effective strategy at reducing the number and activation state of tissue‐infiltrated neutrophils, such an intervention may also affect the recruitment of tissue healing immune cells such as monocytes and M2 macrophages.

## Discussion

The migration of neutrophils from the blood stream into inflamed tissues is a tightly regulated process essential for the rapid development of an effective immune response against injury and invading pathogens. This phenomenon, extensively studied during physiological responses in healthy tissues 34, 35, 38, is also a key element in the development of many acute and life‐threatening inflammatory pathologies 39, 40, such as I/R injury 11, 12, 13, 15. Several therapeutic strategies have been tested both in preclinical and clinical studies of inflammatory conditions to inhibit neutrophil recruitment and/or functions through the use of natural or synthetic (e.g. sivelestat) inhibitors of NE 26, 41. The use of sivelestat, in particular, confirmed the potential of targeting NE for the treatment of disorders such as acute lung injury 42, complications arising from myocardial surgery 43, organ transplantation 44 and several model of I/R injury 45, 46, 47. However, at present the specific role(s) of NE in I/R injury remains poorly understood. To address this issue, here we have comprehensively studied the role of NE in neutrophil migration in multiple murine models of I/R injury using both genetic deletion and pharmacological inhibition. Our findings provide univocal evidence for a vital and non‐redundant role for NE in mediating neutrophil tissue infiltration post‐I/R and categorically identify breaching and remodeling of the venular BM as a key site of action of NE in this process. However, our results also indicate that suppressing NE can lead to reduced tissue infiltration of wound healing immune cells, highlighting a need for a better understanding of the mechanisms of actions of NE inhibitors.

Early studies suggested that NE was a key candidate for the regulation of neutrophil recruitment at sites of I/R injury, although such works failed to fully establish the associated mechanisms 48. NE can potentially impact the migration of neutrophils through blood vessel walls at multiple levels. For example, NE can cleave cell adhesion (e.g. ICAM‐1) 49 and junctional (e.g. JAM‐C) 50 molecules present on the endothelium, proinflammatory cell surface receptors (e.g. TLR4, PAR‐2) 51, 52 and components of the venular BM (e.g. elastin, laminins) 53. NE can also regulate the bioactivity and/or bioavailability of numerous proinflammatory cytokines (TNF) and chemokines (IL‐8) 54, 55. Here we studied NE in the context of I/R injury as induced in the myocardium, kidneys, mesentery and cremaster muscles. In all models, a dramatic suppression of tissue infiltration of neutrophils was noted, with some indications of concomitant inhibition of acute tissue injury. Similar results were obtained using an NE inhibitor, observations that are in agreement with previously published works using different models of I/R injury 45, 46, 47 and confirming the efficacy of sivelestat in the treatment of perioperative acute inflammatory responses in the clinic 42, 43. In sharp contrast, the use of NE‐deficient mice has led to conflicting and varied results regarding the functions of this protease in neutrophil infiltration, questioning the efficacy and specificity of existing NE inhibitors. Several reports have shown that NE^−/−^ mice can exhibit a normal neutrophil transmigration response in models of bacterial infections 29 or in reductive experimental models of tissue inflammation as induced by bacterial derived products (LPS), cytokines (TNF, IL‐1β) 27, 36 or neutrophil chemoattractants (LTB_4_) 56. Such conflicting results may suggest the existence of compensatory mechanisms in NE^−/−^ mice as mediated via the actions of other serine proteases (e.g. PR3) with similar functions to those noted with NE. In support of such a possibility we have previously shown that a broad‐spectrum serine protease inhibitor can suppress IL‐1β‐induced neutrophil transmigration in both WT and NE^−/−^ mice 56. Divergent impacts of NE deficiency could also be attributed to differing mechanisms of action of NE in distinct inflammatory scenarios. For example, the suppression of neutrophil recruitment in NE‐deficient mice within a zymozan‐induced peritonitis model was aligned with reduced generation of the proinflammatory chemokines CXCL1 and CCL3 27. Of note, within the cremaster muscle I/R model employed here, although significant suppression of neutrophil recruitment was observed in NE^−/−^ animals, this was not mediated through reduced levels of endogenous chemokines (e.g. CXCL1 or CCL2; data not shown). Overall, considering the broad substrate specificity of NE and the wide range of stimuli that can induce its release and/or cell surface expression on neutrophils, it is not inconceivable that NE shows different functions and efficacy in regulating neutrophil trafficking as governed by the nature and severity of the inflammatory trigger.

To investigate what stage of neutrophil trafficking NE supported post‐I/R, we analyzed the dynamics of neutrophil responses in I/R‐injured cremaster muscles and mesenteric tissues of WT and NE^−/−^ mice by IVM. These studies identified a selective role for NE in mediating neutrophil breaching of venular walls. Interestingly, adoptive transfer of neutrophils deficient in NE into WT mice led to suppression of neutrophil migration, providing direct evidence for the ability of neutrophil‐derived NE in supporting neutrophil trafficking in a cell‐autonomous manner. In addition to proteases, previous studies have shown that neutrophil‐derived LTB_4_ can act in a feed‐forward manner to support a collective directional motility phenomenon within interstitial tissues known as swarming 57. As well as providing chemotactic cues, cell‐autonomous pathways can regulate neutrophil polarity and migration, such as that reported for neutrophil‐derived ATP and its hydrolyzed form, adenosine 58, and neutrophil‐expressed junctional adhesion molecule‐A (JAM‐A) 59.

Having identified breaching of venular walls as the site of arrest of NE‐deficient neutrophils, detailed analysis of I/R‐injured tissues indicated that NE^−/−^ neutrophils were unable to penetrate the vascular BM. The latter is a critical step in neutrophil breaching of the venular wall and subsequent migration into the interstitial tissue 35. Disruption of venular BM following neutrophil transmigration was first demonstrated *in vitro* 60, although the associated mechanisms remained elusive for many years. Significant insight to this phase of neutrophil trafficking was provided by our previous works where we identified regions within the venular BM that exhibit lower deposition of certain matrix proteins, such as collagen IV and laminins 36, 37. Functionally, these regions are the preferred sites of neutrophil egress through the venular BM and are remodeled by transmigrating neutrophils in terms of their size and protein content upon cytokine‐ and chemokine‐induced inflammation in a strictly neutrophil‐dependent manner 36, 37. These specialized venular wall regions termed matrix protein LERs are now accepted as a key element of neutrophil trafficking 61, 62, 63. In the current study we noted that NE‐mediated neutrophil migration through venular walls involves remodeling of the LERs. These findings are supported by *in vitro* studies showing that NE can cleave ECM proteins such as laminin 53 and collagen 64. In mediating breaching of the venular BM, it is plausible to consider that NE achieves this through localized expression on the cell surface of migrating neutrophils. Indeed, NE can be expressed at the cell surface of neutrophils upon stimulation 56 through its binding to negatively charged glycoproteins 65 or via its interaction with the leukocyte integrin MAC‐1 50, 66. In the present study, the use of an NE‐specific fluorescent activatable substrate indicated the association of NE activity with neutrophils within the abluminal aspect of the vessel wall of cremaster muscles. These findings support the concept that membrane‐bound NE is critical in facilitating NE‐mediated functions as it renders the enzyme resistant to endogenous inhibition 67. Another example of this is the role of NE in supporting the aberrant mode of neutrophil transendothelial migration, neutrophil reverse transendothelial migration, i.e. movement within endothelial cell junctions in an abluminal to luminal direction 68. This junctional reverse motility response of neutrophils was most pronounced following I/R injury 68 and was mechanistically linked with NE‐mediated cleavage of the endothelial cell tight junctional molecule JAM‐C via its binding to neutrophil Mac‐1 *in vivo* 50. In the present study, we observed that in addition to be being cell surface expressed, NE was also released within the venular BM during neutrophil extravasation. Interestingly, the remodeling of LERs in the cremaster muscle and mesenteric I/R injury models was associated with the presence of a subpopulation of tissue‐infiltrated neutrophils that were immunostained for laminin. Collectively these results suggest that neutrophils can remodel the venular BM LERs through localized cell surface expression of NE and/or localized release of NE within the venular BM. The former could lead to neutrophils carrying fragments of venular BM laminin, possibly via their cell surface expression of laminin‐binding receptor α6β1 integrin, as we have previously described 69, 70.

Having found that functional blockade of NE retains neutrophils within venular walls in I/R‐stimulated tissues, and considering the relevance of these findings to future therapeutic use of NE blockers, we sought to investigate if such a vascular retention phenomena could have detrimental vascular/tissue effects. In addressing this notion, the activation state of tissue‐infiltrated neutrophils and vascular permeability in WT and NE^−/−^ mice were quantified. These studies showed reduced neutrophil activation, as measured through ROS generation, and vascular leakage in NE^−/−^ mice, suggesting that retention of potentially activated neutrophils within the venular wall does not impede vascular barrier integrity. However, in assessing the resolution phase of I/R injury, we noted that NE genetic deficiency was associated with reduced recruitment of monocytes and M2 macrophages. These findings are in line with the notion that neutrophil diapedesis can mediate monocyte migration 71, 72 and can promote macrophage polarization towards a prohealing phenotype 73.

Collectively, our findings demonstrate a selective and non‐redundant role for NE in I/R‐induced neutrophil migration through venular walls as mediated via the remodeling of the venular BM. However, although the results suggest that NE inhibitors may be useful strategies in suppressing acute neutrophil‐mediated tissue damage post‐I/R, the findings also raise caution for the use of such drugs as blockade of neutrophil migration may compromise the tissue repair process via inhibiting recruitment of prohealing immune cells.

## Author contributions statement

M‐BV designed and performed most experiments, analyzed data and contributed to the writing of the manuscript; GL designed and performed the myocardial I/R experiments and mesenteric I/R IVM; AW designed and performed the cremaster muscle I/R IVM; LL assisted with image acquisition and analysis of myocardial I/R experiments; NP, RDP and SC designed and performed the mesenteric I/R experiments and data analysis. SN provided overall project supervision, contributed to the design of experiments and the writing of the manuscript.

**SUPPLEMENTARY MATERIAL ONLINE**

**Supplementary materials and methods**

**Supplementary figure legends**

**Figure S1.** Assessment of renal dysfunction following kidney I/R injury

**Figure S2.** Assessment of blood neutrophils, vessel diameter and hemodynamics of post‐capillary venules in WT and NE^−/−^ animals

**Figure S3.** Pharmacological inhibition of NE blocks the migration of neutrophils at the level of the BM post‐I/R injury

**Figure S4.** Specificity of a new rabbit anti‐mouse NE Ab

**Figure S5.** Assessment of blood monocytes of WT and NE^−/−^ animals following I/R injury

**Movie S1.** Brightfield IVM of the WT cremaster muscle subjected to I/R injury. The movie captures the development of the inflammatory response (leukocyte recruitment) of a post‐capillary venule from the cremaster muscle of a WT mouse subjected to ischemia (30 min) and reperfusion (120 min) injury as recorded by brightfield IVM. Red blood cell velocity was measured with an optical Doppler velocimeter (black dots). Quantification of the inflammatory response is shown in Figure 3A.

**Movie S2.** Brightfield IVM of the NE^−/−^ cremaster muscle subjected to I/R injury. The movie captures the development of the inflammatory response (leukocyte recruitment) of a post‐capillary venule from the cremaster muscle of a NE^−/−^ mouse subjected to ischemia (30 min) and reperfusion (120 min) injury as recorded by brightfield IVM. Red blood cell velocity was measured with an optical Doppler velocimeter (black dots). Quantification of the inflammatory response is shown in Figure 3A.

**Movie S3.** Brightfield IVM of the mesenteric tissue subjected to I/R injury. The movie captures the leukocyte inflammatory response of post‐capillary venules from the mesentery of WT and NE^−/−^ mice at 90 min post‐reperfusion as recorded by brightfield IVM. Quantification of the inflammatory response is shown in Figure 3B.

## Supporting information


**Supplementary materials and methods**

**Supplementary figure legends**
**Figure S1. Assessment of renal dysfunction following kidney ischemia/reperfusion injury (I/R injury).** WT and NE^−/−^ mice were subjected to bilateral renal ischemia for 30 min followed by a 24 h reperfusion period. (**A**) H&E staining of kidney sections showing the presence of PMNs (yellow arrows) in the interstitium of the kidney of a WT mice subjected to I/R injury (x40 objective magnification). Bar = 20 μm. The levels of creatinine (**B**) and aspartate aminotransferase (**C**) in mouse plasma were measured as biochemical markers of renal dysfunction subsequent to sham‐operation (WT) or renal ischemia/reperfusion injury (WT and NE^−/−^ animals). Data represent mean ± SEM. *** P < 0.001 (5 mice per group) for comparison between I/R versus sham operated animals; and ## P < 0.01, ### P < 0.001 for comparison between WT and NE^*−/−*^ mice as indicated by lines.
**Figure S2. Assessment of blood neutrophils, vessel diameter and hemodynamics of postcapillary venules in WT and NE** ^**−/−**^ **animals.** The cremaster muscles or mesentery of WT and NE^−/−^ Mice were subjected to ischemia/reperfusion (I/R) injury (30 min/2 h for the cremaster muscles or 35 min/90 min for the mesentery) for analysis of leukocyte responses by bright‐field intravital microscopy and flow cytometry. (**A**) Percentage of circulating neutrophils (of total leukocyte counts) in the blood of animals subjected to I/R of the cremaster muscles (left panel) or mesentery (right panel) and as analyzed by flow cytometry. (**B**) Diameters of post‐capillary venules of animals subjected to I/R of the cremaster muscles (left panel) or mesentery (right panel) as measured by bright‐field intravital microscopy (**C**) Wall shear rate within post‐capillary venules of animals subjected to I/R of the cremaster muscles (left panel) or mesentery (right panel) as measured by bright‐field intravital microscopy. Data represent means ± SEM per mouse. * P < 0.05, ** P < 0.01 (at least 5 mice per group) for comparison between I/R versus sham operated animals.
**Figure S3. Pharmacological inhibition of neutrophil elastase blocks the migration of neutrophils at the level of the basement membrane post I/R injury.** Leukocyte migration responses in the cremaster muscle of WT mice subjected to I/R injury were investigated by intravital and confocal microscopy. To inhibit NE, mice received an intravenous bolus injection of the NE‐specific inhibitor ONO‐5046 (sivelestat) followed by a continuous infusion of this inhibitor (50 mg/kg/h) before the induction of ischemia. Control animals received saline. Leukocyte adhesion (**A**) and extravasation (**B**) responses were quantified at regular intervals for 120 min from the start of the reperfusion period. (**C**) At the end of the experiment, tissues were harvested, fixed and immunostained with fluorescent antibodies against neutrophils (MRP14), endothelial cells (CD31) and the basement membrane (Laminin‐α5) prior to the visualization of the vessels by confocal microscopy. The number of neutrophils present within the venular basement membrane was quantified for each group of animals. (**D**) Quantification of the size of the basement membrane low expression regions (LERs) from cremaster postcapillary venules of mice subjected to I/R injury and/or treated with the NE inhibitor ONO‐5046. Data are from at least 5 mice per group and are presented as mean ± SEM. * P < 0.05, ** P < 0.01, *** P < 0.001, **** P < 0.0001 for comparison between I/R versus sham operated animals; and ## P < 0.01, #### P < 0.0001 for comparison between ONO‐5046 and saline‐treated animals as indicated by lines.
**Figure S4. Specificity of a new rabbit anti‐mouse NE antibody.** (**A**) Blood leukocytes from WT and NE^−/−^ animals were immunostained for NE with a rabbit anti‐mouse NE antibody (green) and neutrophils and rat anti‐mouse MRP14 conjugated with Alexa Fluor 555 (blue) or Alexa Fluor 647 (red) fluorochromes for WT or NE^−/−^ cells, respectively. The nuclei of the leukocytes were revealed with DAPI staining. The images show that WT but not NE^−/−^ neutrophils, exhibit a positive staining for NE in the cytoplasm but not the nucleus of the cell. (**B**) Cremaster muscles of WT and NE^−/−^ mice subjected to I/R injury were harvested, fixed and immunostained for neutrophils (MRP14, blue), pericytes (α‐SMA, green) and neutrophil elastase (red) prior to imaging by confocal microscopy. The images are representative of a WT (left panels) or NE^−/−^ (right panels) mouse post‐capillary venule showing the expression of NE by WT, but not NE^−/−^ neutrophils. A 5% opacity filter on the MRP14 and α‐SMA channels was applied to better visualize NE expression within neutrophils in the bottom images. Images are representative pictures from n = 3 independent experiments. Bar = 10 μm.
**Figure S5: Assessment of blood monocytes of WT and NE** ^**−/−**^ **animals following I/R injury.** The cremaster muscles of WT and NE^−/−^ mice were subjected to 30 min of ischemia followed by a 20 h reperfusion period. The graph shows the quantification of the percentage of circulating neutrophils (of total leukocyte counts) in the blood of animals subjected to I/R of the cremaster muscles (left panel) or mesentery (right panel) and as analyzed by flow cytometry.

**Movie S1. Bright‐field intravital microscopy of the NE** ^**−/−**^ **cremaster muscle subjected to ischemia/reperfusion injury**. The movie captures the development of the inflammatory response (leukocyte recruitment) of a post‐capillary venule from the cremaster muscle of a WT mouse subjected to ischemia (30 min) and reperfusion (120 min) injury as recorded by bright‐field intravital microscopy. Red blood cell velocity was measure with an optical Doppler velocimeter (black dots). Quantification of the inflammatory response is shown in Figure 3A.

**Movie S2. Bright‐field intravital microscopy of the NE** ^**−/−**^ **cremaster muscle subjected to ischemia/reperfusion injury.** The movie captures the development of the inflammatory response (leukocyte recruitment) of a post‐capillary venule from the cremaster muscle of a NE^−/−^ mouse subjected to ischemia (30 min) and reperfusion (120 min) injury as recorded by bright‐field intravital microscopy. Red blood cell velocity was measure with an optical Doppler velocimeter (black dots). Quantification of the inflammatory response is shown in Figure 3A.

**Movie S3. Bright‐field intravital microscopy of the mesenteric tissue subjected to ischemia/reperfusion injury.** The movie captures the leukocyte inflammatory response of post‐capillary venules from the mesentery of WT and NE^−/−^ mice at 90 min post reperfusion as recorded by bright‐field intravital microscopy. Quantification of the inflammatory response is shown in Figure 3B.
