# Supplementary material for: Neutrophil elastase plays a non‐redundant role in remodeling the venular basement membrane and neutrophil diapedesis post‐ischemia/reperfusion injury
Source: J Pathol. 2019 Mar 22;248(1):88–102. doi: 10.1002/path.5234 (PMC6850085; doi:10.1002/path.5234)
Supplement: Supplementary file 1 — Supplementary materials and methods [file PATH-248-88-s005.docx]

**Neutrophil elastase plays a non-redundant role in remodeling the venular basement membrane and supporting neutrophil diapedesis post ischemia/reperfusion injury*.***

Voisin M-B *et al*. *J Pathol* DOI: 10.1002/path.5234

**Supplementary Materials and Methods**

*Reagents/antibodies*

Tyrode’s salt, FCS, paraformaldehyde (PFA), EDTA, Triton X-100, collagenase IV, DNase I, rabbit purified IgG, anti-mouse α-SMA Ab (clone 1A4), PKH26 and Evan’s Blue were all purchased from Sigma-Aldrich (Poole, Dorset, UK). Mouse cytokine and chemokine TNF, IL-1β, CXCL1 and CCL2 ELISA kits were from R&D Systems (Abingdon, UK). Alexa Fluor mAb labeling kits and Alexa Fluor fluorescently labeled secondary Abs, and CountBright™ Absolute Counting Beads were from Invitrogen (Paisley, UK). Anti-mouse Abs against CD31 (clone 390), CD11b (Mac-1, cloneM1/70) and CD115 (clone AFS98), CD45 (clone 30-F11), Ly6G (clone 1A8), F4/80 (cloneEMR1), CD206 (clone MR6F3) and the isotype controls IgG2b and IgG2a were purchased from eBiosciences (Hatfield, UK) and/or Biolegend (London, UK). Anti-MRP-14 [74] (clone 2B10) was a gift from Dr N Hogg (Cancer Research UK, London, UK). Rabbit anti-mouse NE Ab was generated by Eurogentec (Belgium) and specificity of staining was confirmed in both blood and tissue-infiltrated neutrophils of WT and NE^–/–^ animals (see supplementary material, Figure S4). 123Ecount beads and DHE were purchased from ThermoFisher (Paisley, UK) and NE680FAST was obtained from PerkinElmer (Buckinghamshire, UK).

*Animals*

Male C57BL/6 WT mice (Harlan, Bicester, UK) and age-matched NE knockout (NE^–/–^) mice were used. NE^–/–^ mice (backcrossed on C57BL/6 background for at least nine generations) were generated by targeted gene disruption, as previously detailed [28], and were obtained as a gift from Prof S Shapiro (Harvard Medical School, Boston, MA, USA). No differences in neutrophil blood counts, vessel diameter or hemodynamics (i.e. wall shear rate) were noted between WT and NE^–/–^ animals (see supplementary material, Figure S2). Animals were housed in individually ventilated cages and facilities were regularly monitored for health status. All animal experiments were conducted in accordance with UK Home Office legislations and the number of mice per experimental group was kept to the minimum to reach statistical significance (with a minimal significance level of 0.05% and 80% power) and reproducibility in accordance with NC3R recommendations.

*Treatments*

Anesthetized WT mice were injected via jugular vein cannulation with saline or with sivelestat (ONO-5046) (Biotechne, Abingdon, UK) with an initial dose of 50 mg/kg in a 200 μl bolus preceding the induction of ischemia followed by an infusion of 50 mg/kg in 200 μl/h during the reperfusion period, as per previous works [56].

*I/R injury of the heart*

Anasthetized animals (150 mg/kg ketamine, 7.5 mg/kg xylazine, i.p.) were subjected to a tracheotomy for artificial ventilation (Harvard Apparatus, AH 40-1000). The tidal volume of the respirator was set at 1 ml/min, with the rate set at 100 strokes/min, and was supplemented with 100% oxygen. After completion of the surgical procedures, mice were allowed to stabilize for 30 min before ligation of the left anterior descending coronary artery (LADCA) was performed using a 6/0 silk suture (Ethicon, W593 7/0 BV1, Edinburgh, UK). Sham-operated animals received the same surgical procedure without completing the full ligation of the LADCA. Ischemia was confirmed by the appearance of hypokinesis and pallor distal to the occlusion. After a 25 min period of myocardial ischemia the clip was removed to allow tissue reperfusion (visually confirmed) for 2 h. At the end of the experiment, the mice were killed; hearts were removed and ‘snap’ frozen with liquid nitrogen before being processed for cryosectioning and confocal microscopy analysis.

*I/R injury of the kidneys*

Mice were anesthetized for the duration of the surgical procedures (ketamine 125 mg/kg, i.p.). Animals were subjected to bilateral renal ischemia for 30 min following abdominal laparotomy, during which the renal arteries and veins were occluded using micro-aneurysm clamps, as previously described [75]. After the removal of renal clamps, the incision was sutured and mice were allowed to recover from anesthesia for 24 h with analgesia (buprenorphine 10 mg/kg s.c.). Sham-operated mice underwent identical surgical procedures without the use of micro-aneurysm clamps. At the end of the experiments, the mice were humanely sacrificed, blood samples were taken for serum analysis and kidneys were collected for histological section analysis.

*IVM and induction of cremasteric I/R injury*

IVM was used to directly observe leukocyte responses within mouse cremaster venules, as previously detailed [32]. In brief, mice were anesthetized (125 mg/kg ketamine, 12.5 mg/kg, xylazine, i.p). The cremaster muscle was surgically exteriorized on to a purpose-built microscope stage and was kept warm and moist by continuous application of warmed Tyrode’s balanced salt solution. Tissue ischemia was induced by placing an artery clamp at the proximal end of the pinned cremaster tissue for 30 min. Reperfusion was initiated by releasing the clamp and restoring blood flow for up to 2 h. Leukocyte responses were observed on an upright brightfield microscope (Carl Zeiss, Welwyn Garden City, UK). Firmly adherent cells were those remaining stationary for 30 s or longer within a given 500 µm segment of the endothelial surface of the lumen of post-capillary venules (20–40 µm in diameter). Extravasated leukocytes were quantified within 50 µm on either side of the 500 µm vessel segment in the perivenular tissue. Red blood cell centerline velocity was measured in post-capillary venules with an optical Doppler velocimeter (Microcirculation Research Institute, Texas A&M University, Dallas, TX, USA) and venular wall shear rate was determined based on the Newtonian definition: wall shear rate = 8000 [(red blood cell velocity/1.6)/venular diameter]. At the end of the experiment, mice were humanely killed, tissue removed and fixed in 4% PFA or 100% ice-cold methanol for 30 min for immunostaining purposes.

*IVM and induction of I/R injury of the mesenteric tissue*

Anesthetized mice (150 mg/kg ketamine, 7.5 mg/kg xylazine, i.p.) were placed in supine position on a heating pad (37 ºC) for maintenance of body temperature. Mesenteric ischemia was induced with a micro-aneurysm clip (Harvard Apparatus), clamping the superior mesenteric artery for 35 min. The clip was then removed and reperfusion allowed for 90 min. Sham-operated animals underwent the same surgical procedure except for the clamping of the superior mesenteric artery. The mesenteric vascular bed was exteriorized, placed on a purpose-built stage of an upright brightfield microscope (Zeiss Axioskop “FS”). Mesenteries were superfused with warmed (37 ºC) bicarbonate buffer solution. A 5-min equilibration period preceded the ischemia. Analysis of leukocyte–endothelium interactions was made in one to three randomly selected post-capillary venules (20–40 µm in diameter, 100 µm in length) for each mouse 90 min post-reperfusion. Leukocyte adhesion (stationary position of the cell for 30 s or longer) was quantified along a 100 µm vessel length. Leukocyte extravasation response was quantified within 50 µm on either side of the 100 µm vessel segment in the perivenular tissue. Red blood cell centerline velocity was measured in post-capillary venules with an optical Doppler velocimeter (Microcirculation Research Institute) and venular wall shear rate was determined based on the Newtonian definition: wall shear rate = 8000 [(red blood cell velocity/1.6)/venular diameter]. At the end of the reperfusion and analysis period, mice were humanely killed and mesenteric tissues removed and fixed in 4% PFA for 30 min prior to immunostaining for observation by confocal fluorescence microscopy.

*Determination of renal injury and dysfunction*

Blood samples were collected via cardiac puncture into S/1.3 tubes containing serum gel (Sarstedt, Germany). The samples were centrifuged (6000 × *g* for 3 min). Serum levels of creatinine and aspartate aminotransferase were quantified by ELISA (R&D Systems).

*Histological evaluation of renal injury*

Kidneys were cut in a sagittal section into two halves, fixed in 10% (w/v) PFA at room temperature for 1 week. After dehydration using graded ethanol, tissues were embedded in Paraplast (Sherwood Medical, Mahwah, NJ, USA) and cut into 8 µm thick sections mounted on glass slides. Sections were then deparaffinized with xylene, stained with H&E and viewed under a light microscope (Dialux 22, Leitz, Milan, Italy). For the histopathological score, 100 intersections were examined for each kidney and a score from 0 to 3 was given for each tubular profile involving an intersection: 0, normal histology; 1, tubular cell swelling, brush border loss, nuclear condensation, with up to one third of the tubular profile showing nuclear loss; 2, as for score 1, but greater than one third and less than two thirds of tubular profile show nuclear loss; 3, more than two thirds of tubular profile shows nuclear loss. The total score for each kidney was calculated by addition of all 100 scores with a maximum score of 300. All the histological studies were performed in a blind manner [76]. The total number of infiltrating leukocytes (e.g. neutrophils and mononuclear cells) in cortical interstitial spaces was assessed quantitatively by counting the number of PMNs in high-power fields using ×20 or ×40 objectives based on the morphology of the nuclei of H&E-stained sections (see supplementary material, Figure S1A).

*Adoptive cell transfer experiments*

To investigate the behavior specifically of WT or NE^–/–^ neutrophils during their migration through the venular wall, adoptive transfer experiments were performed. For this purpose, bone marrow leukocytes from WT or NE^–/–^ donor mice were harvested from the femur and tibia. Cells were then sieved and counted, resuspended in PBS and incubated with the membrane labeling fluorescent indicator PKH26 according to the manufacturer’s recommendations (Sigma) at 37 °C for 30 min. Cells were then injected i.v. into WT or NE^–/–^ recipient animals via the tail vein (10^7^ cells/mouse) prior to being subjected to I/R injury of the cremaster muscles as detailed above. At the end of the reperfusion period, the cremaster muscles were exteriorized, fixed and immunostained as detailed below for observation by confocal fluorescent microscopy. Blood samples were also taken from cardiac puncture and the number of circulating PKH26+ cells was analyzed by flow cytometry. The results are shown as the number of transmigrated PKH26 cells in 0.02 mm^2^ of tissue area per % of PHK26+ cells in the blood in order to normalize the results for the number of blood recirculating cells following the adoptive transfer.

*Analysis of tissues by immunofluorescence labeling and confocal microscopy*

Detection of neutrophils in the ischemic region of the heart was performed on OCT-embedded heart tissue sections. In brief , sections (30 µm thick) were fixed for 10 min with methanol, permeabilized and blocked in PBS supplemented with 10% FCS, 10% GS and 0.5% Triton X-100 for 1 h and then incubated in PBS + 10% FCS overnight at room temperature with a rabbit anti-mouse collagen IV polyclonal Ab, and fluorescently labeled rat anti-mouse CD31 and a rat anti-mouse MRP-14 Ab at a concentration of 10 µg/ml to label the endothelium and neutrophils, respectively. Sections were then washed in PBS and incubated with Alexa Fluor-488-labeled goat anti-rabbit for 2 h at room temperature. Tissue sections were then visualized using confocal microscopy (Zeiss LSM5 Pascal) using 10× (NA:0.25) or 20× (NA:0.50) objectives. Images were subsequently analyzed with IMARIS analysis software (Bitplane, Switzerland). To reconstruct the entire heart sections, serial images were taken and reassembled using Photoshop CS3. Fluorescence intensity quantification was carried out using IMARIS software using the same settings for all groups.

Detection of neutrophils into the cremaster muscles and mesenteric tissue following I/R injury was performed by immunostaining whole-mount tissues and visualization by confocal microscopy. In brief, PFA-fixed and permeabilized tissues (PBS containing 10% normal goat serum, 10% FCS, 5% mouse serum and 0.5% Triton X-100 for 2 h at room temperature) were immunostained for neutrophils (rat anti-mouse MRP-14 mAb) and the perivascular BM (rabbit anti-mouse LNα-5 mAb, gift from Prof Tanaka) in PBS + 10% FCS overnight at 4 °C. MRP-14 is an intracellular protein highly abundant in neutrophils that is well accepted as a neutrophil-specific marker for whole-mount tissue immunostaining and confocal microscopy [74,77]. Following three washes in PBS, tissues were subsequently incubated with specific Alexa Fluor-633-conjugated anti-rat or Alexa Fluor 555-conjugated anti-rabbit secondary Abs (Invitrogen) for 4 h at room temperature in PBS + 10% FCS. In order to stain for endothelial cells, tissues were blocked with rat IgGs for 2 h before adding a rat anti-mouse Alexa Fluor-488-conjugated CD31 mAb. For NE expression, WT and NE^–/–^ tissues were first immunostained against neutrophils (rat anti-mouse MRP-14) and rabbit anti-NE Abs in PBS + 10% FCS overnight at 4 °C. Following three washes in PBS, tissues were subsequently incubated with specific Alexa Fluor-647-conjugated anti-rat- and -555-conjugated anti-rabbit secondary Abs for 4 h at room temperature in PBS + 10% FCS. In order to stain for venular BM, tissues were blocked with rabbit IgGs for 2 h before being incubated with the rabbit anti-mouse pan-laminin Ab directly labeled with Alexa Fluor-488 according to the Ab labeling Zenon kit protocol. All samples were viewed using a Zeiss LSM 5 Pascal laser-scanning confocal microscope (Carl Zeiss Ltd, Welwyn Garden City, UK) with a 40× objective (NA:0.75). All image acquisition parameters for NE expression as quantified by immunostaining and confocal microscopy (e.g. laser power, detector gain voltage and image format) were normalized to NE^–/–^ tissues. In some experiments, the specificity of staining was also confirmed in WT tissues immunostained with an irrelevant rabbit Ig control Ab (not shown). Acquired Z-stack images were used and analyzed for 3D reconstruction of whole vessels of 200 μm length (four to six vessels per tissue) with the image processing software IMARIS allowing accurate visualization of the position of leukocytes relative to the endothelium and the perivascular BM. Mean fluorescent intensity quantification of NE fluorescence was quantified using a mask (isosurface) on the MRP-14 or laminin channels using the same settings throughout the analysis. The area and intensity of matrix protein LERs within the perivascular BM were measured using ImageJ software (NIH & Laboratory for Optical and Computational Instrumentation, University of Wisconsin, Madison, WI, USA) as previously detailed [36,37].

For the visualization of NE enzymatic activity *in vivo*, mice were subjected to non-surgical ischemia using an orthodontic band placed at the top of the scrotum for 35 min followed by 1 h of reperfusion. The NE-fluorescent activatable substrate NE680FAST was injected i.v. (4.8 nmol/mouse) and a non-blocking Alexa Fluor 555-conjugated rat anti-mouse CD31 (2 µg/mouse) was injected intrascrotally at the end of the ischemic period. At the end of the reperfusion, tissues were harvested, fixed and immunostained with Alexa Fluor 488-conjugated anti-MRP-14 Ab as described above. All samples were then viewed using a Leica SP8 laser scanning confocal microscope with a 20× objective (NA:1) and images of post-capillary venules (at least six vessels per tissue) were acquired with the use of sequential scanning of different channels at every 0.5 μm of tissue depth at a resolution of 1024 × 470 pixels in the *x* × *y* plane. This pixel resolution corresponds to a voxel size of 0.24 × 0.24 × 0.5 μm in *x* × *y* × *z*. All Images were subsequently analyzed offline with the 3D reconstructing image processing software IMARIS.

The specificity of the anti-NE Ab staining was also assessed in blood neutrophils by intracellular immunostaining. In brief, blood leukocytes from WT and NE^–/–^ animals were harvested by cardiac puncture, the erythrocytes lysed with ACK buffer solution (150 mm NH_4_Cl, 1 mm KHCO_3_, 0.1 mm EDTA) and the leukocytes from both genotypes were fixed and permeabilized using the BD Cytofix/Cytoperm™ kit according to the reagent protocol. Cells were then incubated with the solution of staining buffer containing 1% CD16/CD32 blocking reagent and the purified rabbit anti-mouse NE Ab overnight at 4 °C. WT or NE^–/–^ cells were also incubated with an Alexa Fluor 555-conjugated or Alexa Fluor 647 rat anti-mouse MRP-14, respectively. Cell were then washed four times prior to incubation with an Alexa Fluor 488-conjugated goat anti-rabbit secondary Ab for 2 h at 4 °C. After four final washes, both WT and NE^–/–^ samples were then mounted on the same slides with DAPI-containing mounting medium prior to visualization using a Zeiss 800 laser scanning confocal microscope (Carl Zeiss) with a 63× objective (NA:1.4). All images were subsequently analyzed offline with the 3D reconstructing image processing software IMARIS.

*Neutrophil and monocyte phenotypic analysis by flow cytometry*

The cremaster muscles of WT and NE^–/–^ were subjected to 35 min of non-surgical ischemia using an orthodontic band followed by 20 h of reperfusion. Blood (200 µl/animal) was collected by cardiac puncture and the cremaster muscles were harvested and digested into a single cell suspension with collagenase and DNase I for 30 min at 37 °C. Cells were fluorescently labeled with conjugated Abs against CD45, Ly6G, CD11b, CD115, CD206, F4/80 (0.2–2 μg/ml, various fluorochromes) and with FC block for at least 30 min at 4 °C. For ROS detection, cells where first preincubated with 1 µm DHE for 15 min at 37 °C prior to the incubation with Abs. CountBright™ Absolute Counting Beads were used to quantify the total number of cells, following manufacturer’s recommendations. Samples were analyzed using a BD LSR-Fortessa (BD Biosciences) and FlowJo analysis software (Treestar, BD Biosciences). Leukocytes were first gated with CD45 and FCS/SSC morphology (excluding cell doublet). Neutrophils were identified as CD45+ CD11b+ Ly6G+, monocytes were identified as CD45+ Ly6G- CD11b+, CD115+, F4/80^low^; and M2 macrophage were identified as CD45+ Ly6G- CD11b^high^, F4/80+, CD206+.

*Vascular leakage assay (Evan’s Blue Miles Assay)*

Vascular leakage was assessed in the mouse cremaster muscle of WT and NE^–/–^ mice following I/R injury. In brief, cremaster muscles were subjected to 35 min of non-surgical ischemia using an orthodontic band followed by 2 h reperfusion. Mice where then injected i.v. with 0.5% Evan’s Blue solution (6 µl/g body weight). Two hours later (i.e. 4 h post-reperfusion), mice were sacrificed and the cremaster muscles were excised and incubated in 100% formamide overnight at 56 °C. The optical density of eluted Evan’s Blue in 50 µl of samples was measured by spectrophotometry at a wavelength of 620 nm. The results are presented as the optical density (OD) of Evan’s Blue leakage [31].

*Statistical analyses*

All data were processed and analyzed using Prism 4 software (GraphPad Inc, San Diego, CA, USA). Statistical significance was assessed by parametric (one-way/two-way ANOVA followed by Newman-Keuls multiple comparison test) and non-parametric (Kruskal-Wallis followed Dunn's multiple comparisons test) tests according to the sample size of the data analyzed. Where only two variables were analyzed, an unpaired *t*-test (Mann-Whitney) was used. *p* < 0.05 was taken as significant. Results are given as mean ± SEM.
