# Supplementary material for: Neutrophil elastase plays a non‐redundant role in remodeling the venular basement membrane and neutrophil diapedesis post‐ischemia/reperfusion injury
Source: J Pathol. 2019 Mar 22;248(1):88–102. doi: 10.1002/path.5234 (PMC6850085; doi:10.1002/path.5234)
Supplement: Supplementary file 2 — Supplementary figure legends Figure S1. Assessment of renal dysfunction following kidney ischemia/reperfusion injury (I/R injury). WT and NE−/− mice were subjected to bilateral renal ischemia for 30 min followed by a 24 h reperfusion period. (A) H&E staining of kidney sections showing the presence of PMNs (yellow arrows) in the interstitium of the kidney of a WT mice subjected to I/R injury (x40 objective magnification). Bar = 20 μm. The levels of creatinine (B) and aspartate aminotransferase (C) in mouse plasma were measured as biochemical markers of renal dysfunction subsequent to sham‐operation (WT) or renal ischemia/reperfusion injury (WT and NE−/− animals). Data represent mean ± SEM. *** P < 0.001 (5 mice per group) for comparison between I/R versus sham operated animals; and ## P < 0.01, ### P < 0.001 for comparison between WT and NE−/− mice as indicated by lines. Figure S2. Assessment of blood neutrophils, vessel diameter and hemodynamics of postcapillary venules in WT and NE −/− animals. The cremaster muscles or mesentery of WT and NE−/− Mice were subjected to ischemia/reperfusion (I/R) injury (30 min/2 h for the cremaster muscles or 35 min/90 min for the mesentery) for analysis of leukocyte responses by bright‐field intravital microscopy and flow cytometry. (A) Percentage of circulating neutrophils (of total leukocyte counts) in the blood of animals subjected to I/R of the cremaster muscles (left panel) or mesentery (right panel) and as analyzed by flow cytometry. (B) Diameters of post‐capillary venules of animals subjected to I/R of the cremaster muscles (left panel) or mesentery (right panel) as measured by bright‐field intravital microscopy (C) Wall shear rate within post‐capillary venules of animals subjected to I/R of the cremaster muscles (left panel) or mesentery (right panel) as measured by bright‐field intravital microscopy. Data represent means ± SEM per mouse. * P < 0.05, ** P < 0.01 (at least 5 mice per group) for comparison between I/R versus s [file PATH-248-88-s004.docx]

**Neutrophil elastase plays a non-redundant role in remodeling the venular basement membrane and supporting neutrophil diapedesis post ischemia/reperfusion injury*.***

Voisin M-B *et al*. *J Pathol* DOI: 10.1002/path.5234

**Supplementary Figures**

**Figure S1**


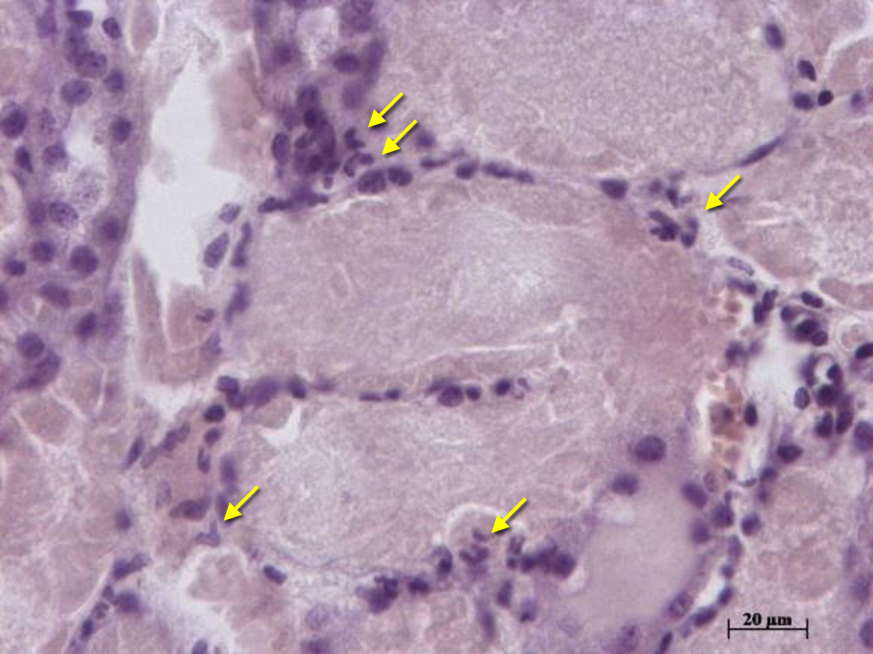


**A**

**C**

**B**

**
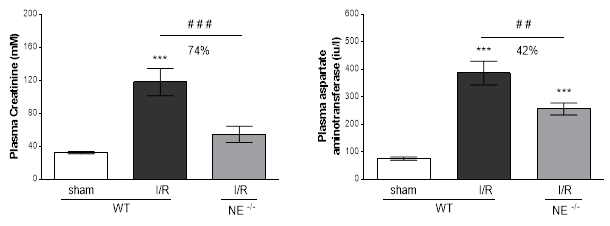
**

**Figure S1. Assessment of renal dysfunction following kidney I/R injury.** WT and NE^–/–^ mice were subjected to bilateral renal ischemia for 30 min followed by a 24 h reperfusion period. (A) H&E staining of kidney sections showing the presence of PMNs (yellow arrows) in the interstitium of the kidney of a WT mice subjected to I/R injury (x40 objective magnification). Bar = 20 µm. The levels of creatinine (B) and aspartate aminotransferase (C) in mouse plasma were measured as biochemical markers of renal dysfunction subsequent to sham-operation (WT) or renal I/R injury (WT and NE^–/–^ animals). Data represent mean ± SEM. ****p* < 0.001 (five mice per group) for comparison between I/R and sham-operated animals; ##*p* < 0.01, ###*p* < 0.001 for comparison between WT and NE*^–/–^* mice as indicated by lines.

**Figure S2**

**Cremaster muscles**

**Mesentery**


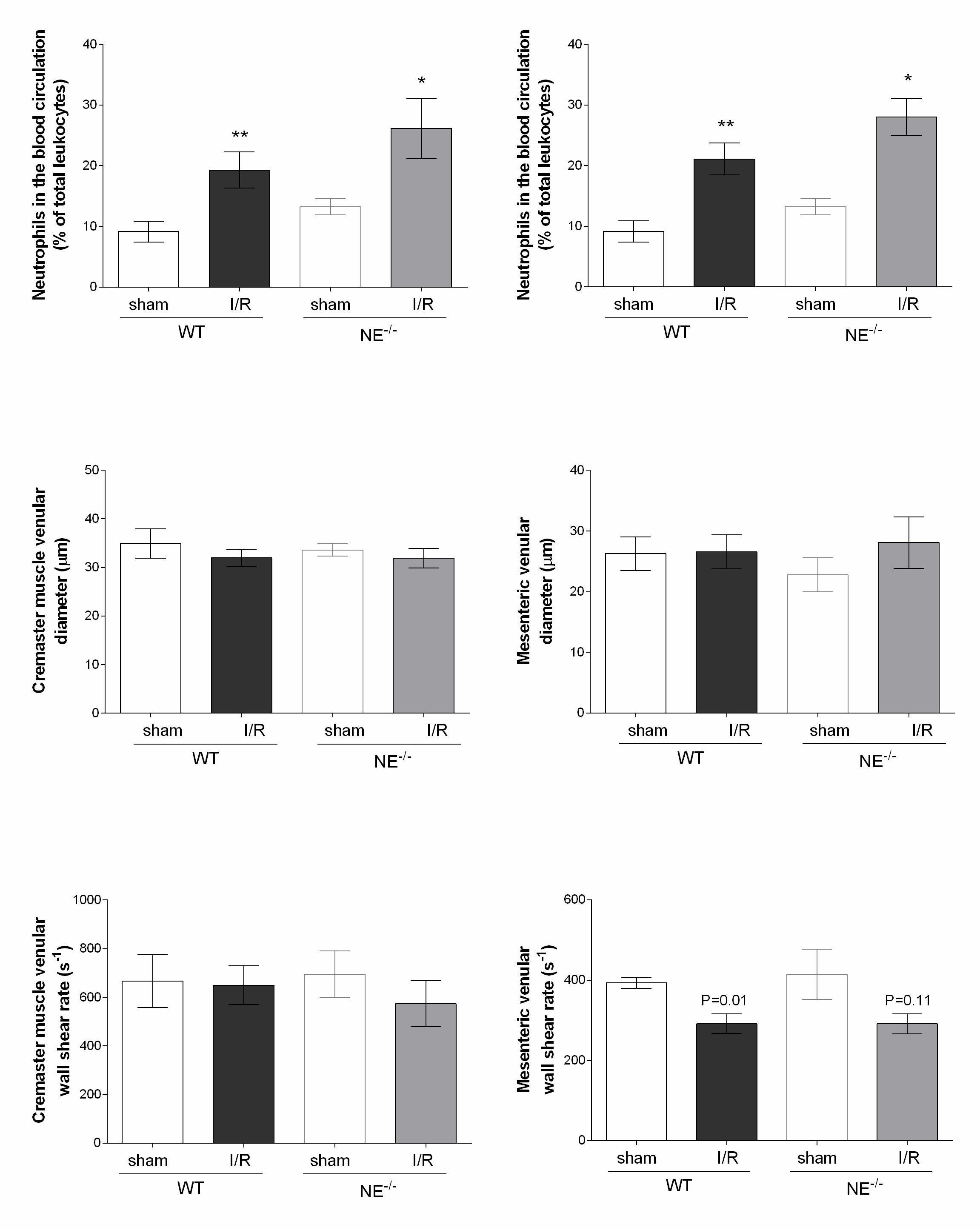


**C**

**B**

**A**

**Figure S2. Assessment of blood neutrophils, vessel diameter and hemodynamics of post-capillary venules in WT and NE^–/–^ animals.** The cremaster muscles or mesentery of WT and NE^–/–^ mice were subjected to I/R injury (30 min/2 h for the cremaster muscles or 35 min/90 min for the mesentery) for analysis of leukocyte responses by brightfield IVM and flow cytometry. (A) Percentage of circulating neutrophils (of total leukocyte counts) in the blood of animals subjected to I/R of the cremaster muscles (left panel) or mesentery (right panel) and as analyzed by flow cytometry. (B) Diameters of post-capillary venules of animals subjected to I/R of the cremaster muscles (left panel) or mesentery (right panel) as measured by brightfield IVM. (C) Wall shear rate within post-capillary venules of animals subjected to I/R of the cremaster muscles (left panel) or mesentery (right panel) as measured by brightfield IVM. Data represent means ± SEM per mouse. **p* < 0.05, ***p* < 0.01 (at least five mice per group) for the comparison between I/R and sham-operated animals.

**Figure S3**

**
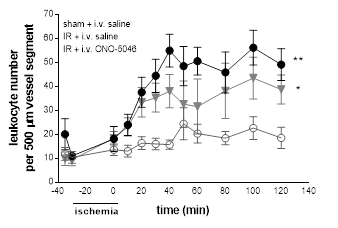

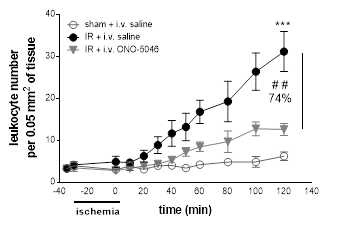
**

**B** Extravasation

**A** Adhesion


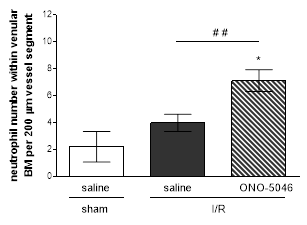
**C D**


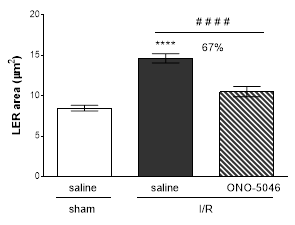


**Figure S3. Pharmacological inhibition of NE blocks the migration of neutrophils at the level of the BM post-I/R injury.** Leukocyte migration responses in the cremaster muscle of WT mice subjected to I/R injury were investigated by IVM and confocal microscopy. To inhibit NE, mice received an i.v. bolus injection of the NE-specific inhibitor ONO-5046 (sivelestat) followed by a continuous infusion of this inhibitor (50 mg/kg/h) before the induction of ischemia. Control animals received saline. Leukocyte adhesion (A) and extravasation (B) responses were quantified at regular intervals for 120 min from the start of the reperfusion period. (C) At the end of the experiment, tissues were harvested, fixed and immunostained with fluorescent Abs against neutrophils (MRP-14), endothelial cells (CD31) and the BM (laminin-α5) prior to the visualization of the vessels by confocal microscopy. The number of neutrophils present within the venular BM was quantified for each group of animals. (D) Quantification of the size of the BM LERs from cremaster post-capillary venules of mice subjected to I/R injury and/or treated with the NE inhibitor ONO-5046. Data are from at least five mice per group and are presented as mean ± SEM. **p* < 0.05, ***p* < 0.01, ****p* < 0.001, *****p* < 0.0001 for the comparison between I/R and sham-operated animals; ##*p* < 0.01, ####*p* < 0.0001 for the comparison between ONO-5046 and saline-treated animals as indicated by lines.

**Figure S4**

**A**

MRP14 (WT neutrophils)

MRP14 (NE^-/-^ neutrophils)

NE DAPI

NE, DAPI

NE alone


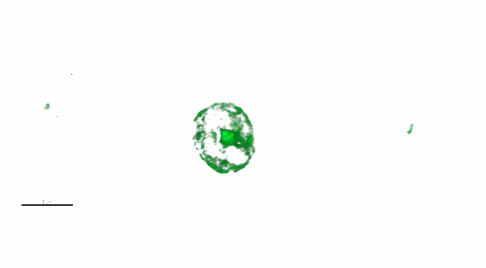

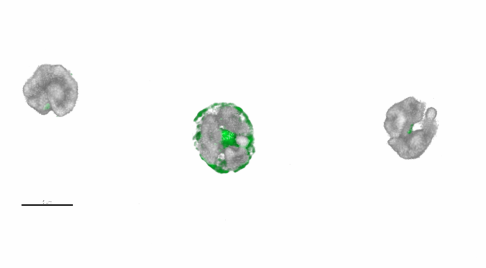

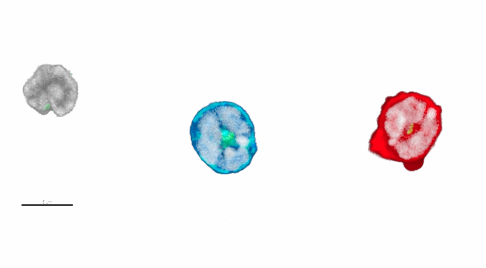


**B**  WT cremaster tissue (I/R) NE^-/-^ cremaster tissue (I/R)


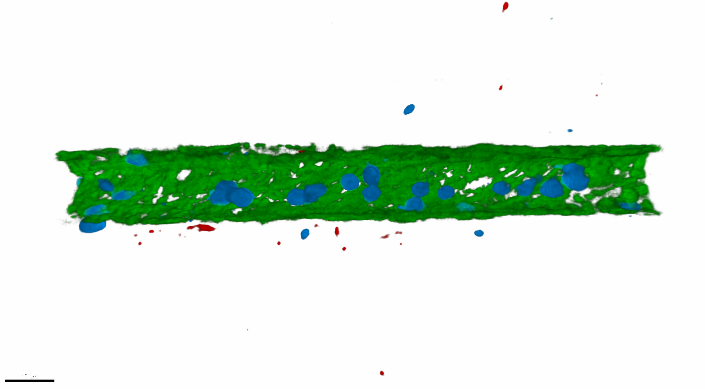

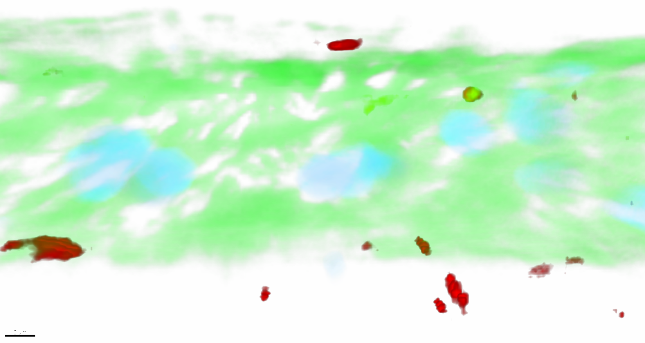

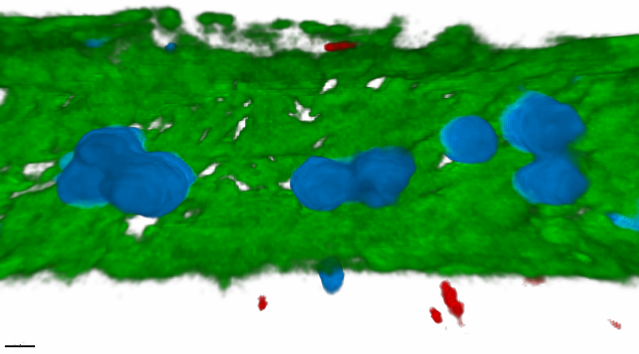

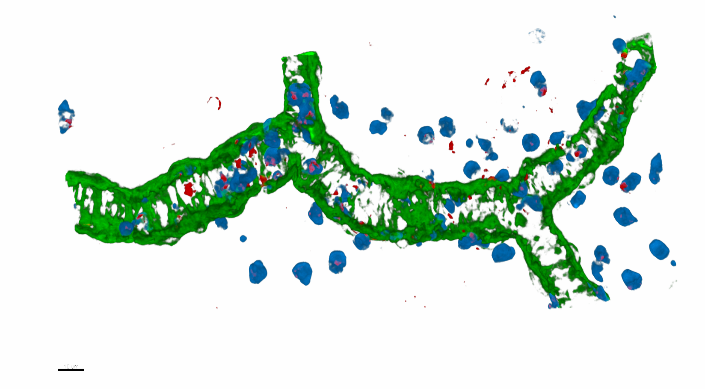

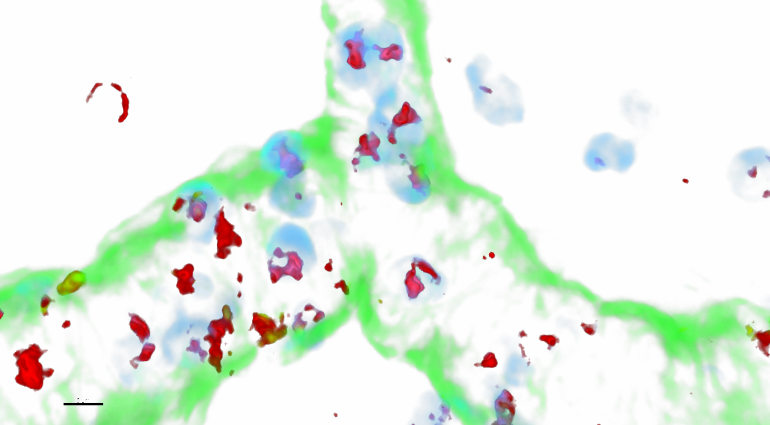

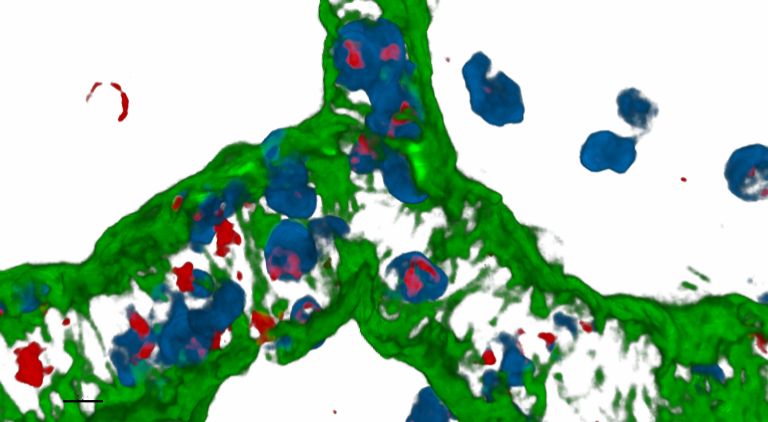


Neutrophils

pericytes

NE

**Figure S4. Specificity of a new rabbit anti-mouse NE Ab.** (A) Blood leukocytes from WT and NE^–/–^ animals were immunostained for NE with a rabbit anti-mouse NE Ab (green) and neutrophils and rat anti-mouse MRP-14 conjugated with Alexa Fluor-555 (blue) or Alexa Fluor-647 (red) fluorochromes for WT or NE^–/–^ cells, respectively. The nuclei of the leukocytes were revealed with DAPI staining. The images show that WT, but not NE^–/–^ neutrophils, exhibit a positive staining for NE in the cytoplasm but not the nucleus of the cell. (B) Cremaster muscles of WT and NE^–/–^ mice subjected to I/R injury were harvested, fixed and immunostained for neutrophils (MRP-14, blue), pericytes (α-SMA, green) and NE (red) prior to imaging by confocal microscopy. The images are representative of a WT (left panels) or NE^–/–^ (right panels) mouse post-capillary venule showing the expression of NE by WT, but not NE^–/–^ neutrophils. A 5% opacity filter on the MRP-14 and α-SMA channels was applied to better visualize NE expression within neutrophils in the bottom images. Images are representative pictures from *n* = 3 independent experiments. Bar = 10 µm.

**Figure S5**

**
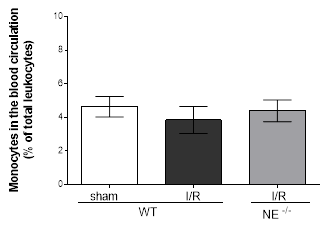
**

**Figure S5. Assessment of blood monocytes of WT and NE^–/–^ animals following I/R injury.** The cremaster muscles of WT and NE^–/–^ mice were subjected to 30 min of ischemia followed by a 20 h reperfusion period. The graph shows the quantification of the percentage of circulating neutrophils (of total leukocyte counts) in the blood of animals subjected to I/R of the cremaster muscles (left panel) or mesentery (right panel) and as analyzed by flow cytometry.
